# Screening for cardiovascular disease risk and subsequent management in low and middle income countries: challenges and opportunities

**DOI:** 10.1186/s40985-015-0013-0

**Published:** 2015-11-25

**Authors:** Pascal Bovet, Arnaud Chiolero, Fred Paccaud, Nick Banatvala

**Affiliations:** 1 Institute of Social and Preventive Medicine (IUMSP), University Hospital Centre, Rue de la Corniche 10, 2013 Lausanne, Switzerland; 2 Noncommunicable Diseases and Mental Health Cluster, World Health Organization, Geneva, Switzerland

**Keywords:** Strengthen Health System, Primary Health Care Level, World Health Assembly, Voluntary Global Target, Main NCDs

## Abstract

**Background:**

Cardiovascular disease (CVD), mainly heart attack and stroke, is the leading cause of premature mortality in low and middle income countries (LMICs). Identifying and managing individuals at high risk of CVD is an important strategy to prevent and control CVD, in addition to multisectoral population-based interventions to reduce CVD risk factors in the entire population.

**Methods:**

We describe key public health considerations in identifying and managing individuals at high risk of CVD in LMICs.

**Results:**

A main objective of any strategy to identify individuals at high CVD risk is to maximize the number of CVD events averted while minimizing the numbers of individuals needing treatment. Scores estimating the total risk of CVD (e.g. ten-year risk of fatal and non-fatal CVD) are available for LMICs, and are based on the main CVD risk factors (history of CVD, age, sex, tobacco use, blood pressure, blood cholesterol and diabetes status). Opportunistic screening of CVD risk factors enables identification of persons with high CVD risk, but this strategy can be widely applied in low resource settings only if cost effective interventions are used (e.g. the WHO Package of Essential NCD interventions for primary health care in low resource settings package) and if treatment (generally for years) can be sustained, including continued availability of affordable medications and funding mechanisms that allow people to purchase medications without impoverishing them (e.g. universal access to health care). This also emphasises the need to re-orient health systems in LMICs towards chronic diseases management.

**Conclusion:**

The large burden of CVD in LMICs and the fact that persons with high CVD can be identified and managed along cost-effective interventions mean that health systems need to be structured in a way that encourages patient registration, opportunistic screening of CVD risk factors, efficient procedures for the management of chronic conditions (e.g. task sharing) and provision of affordable treatment for those with high CVD risk. The focus needs to be in primary care because that is where most of the population can access health care and because CVD programmes can be run effectively at this level.

## Burden of cardiovascular disease and impact in LMICs

Cardiovascular disease (CVD), mainly heart attack and stroke, is the leading cause of premature mortality and morbidity worldwide [1–3]. An estimated 38 million of the 56 million deaths that occurred globally in 2012 were due to noncommunicable diseases (NCDs) (i.e. CVD, cancer, diabetes and chronic respiratory diseases), with CVD accounting for 46 % of NCD deaths. In 2008, 80 % of all deaths from NCDs occurred in low- and middle-income countries (LMICs). The good news is that premature fatal and non-fatal CVD is largely preventable, and feasible cost-effective interventions exist [4, 5], which emphasizes the need to respond to CVD and other leading NCDs in all countries. In most LMICs, the majority of people at high risk of CVD, which largely correspond to those people with hypertension, high blood cholesterol and/or diabetes, are not aware of having these conditions and do not appreciate that these are risk factors for CVD or that these conditions can be controlled with effective management [6, 7]. In addition, many people in LMICs are unaware of the lifestyle behaviours that are associated with increased risk of CVD and other NCDs, such as tobacco use, harmful use of alcohol, unhealthy diet and physical inactivity.

## Strategies to prevent CVD in populations

**Fig. 1:**
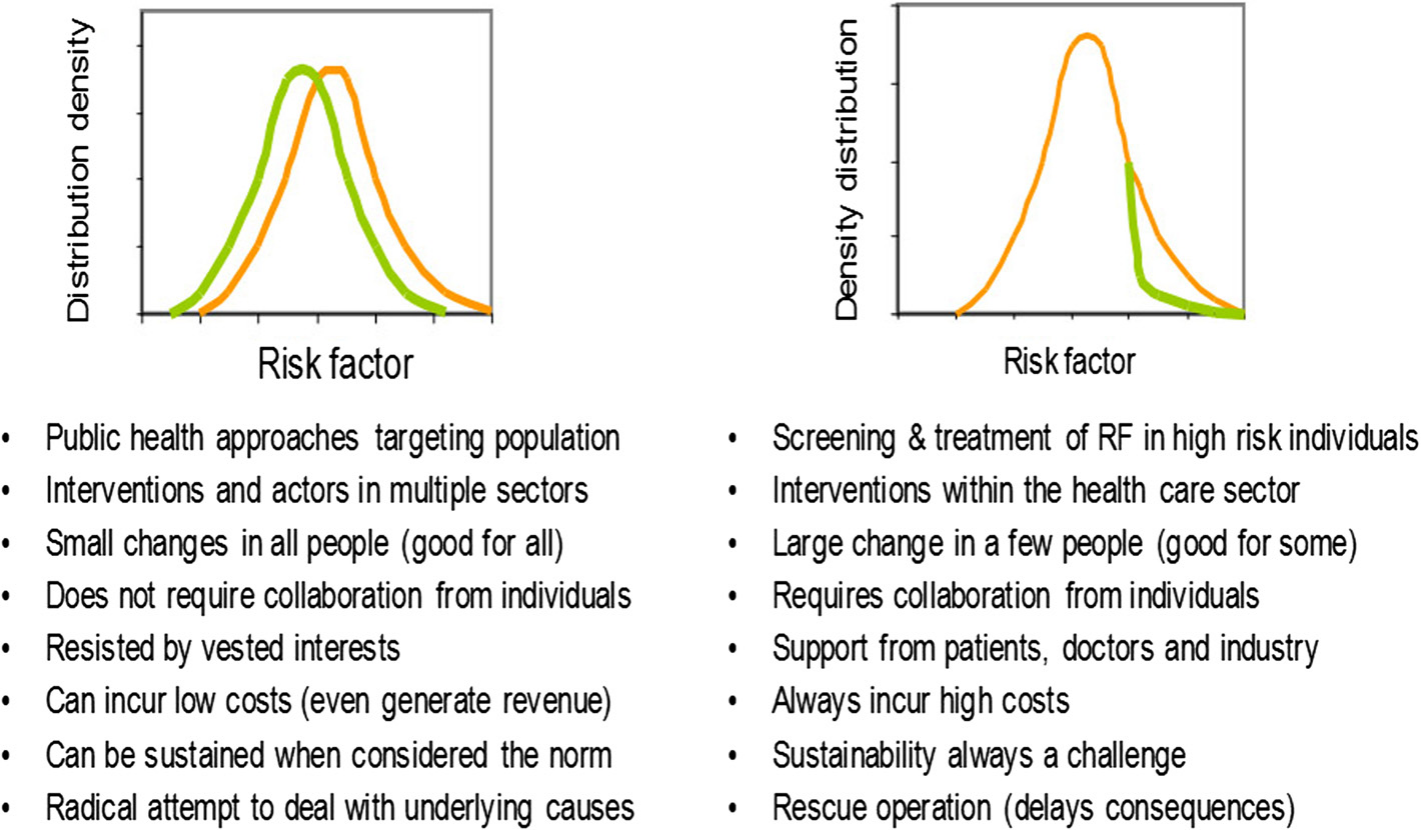
Selected characteristics of the population strategy (left) and high risk strategy (right)

There are two main strategies to prevent CVD: population and high-risk [8]. Advantages and disadvantages of these two strategies are summarized in Fig. 1. Population strategies involve multisectoral interventions to reduce risk factors in the population. They deploy small effects at the individual level (i.e., small reduction in risk factors) and are “good for all” (e.g. tobacco taxation or reduction of salt, sugar and trans-fats in processed foods). The financial cost of their implementation is often low and some interventions can generate substantial revenue (e.g. taxes on tobacco and alcohol). On the other hand, the health and social costs tend to be high when the determinants of diseases are related to profitable production of goods (e.g., tobacco, alcohol or food industries).

High-risk strategies involve health care services; they bring large benefit (i.e. large reduction of some risk factors) to those persons treated, are “good for some”, but require the explicit engagement of individuals (e.g. long-term adherence to medication). The financial cost is often high because management of NCDs often requires life-long clinical medications (e.g. hypertension, dyslipidaemia, diabetes, etc.). These interventions tend to benefit from a broad support by patients, health professionals and government because their effect is clinically apparent and immediate. Nevertheless there are a number of high risk interventions that are cost-effective and feasible.

Screening of individuals at high CVD risk can also be viewed as a strategy to detect sub-clinical CVD (e.g. coronary atherosclerosis, increased artery intima-media thickness, enlarged left myocardial ventricular mass, endothelial artery dysfunction, etc.) and treatment for these persons viewed as a mean to improve the prognosis of such subclinical CVD condition(s). In this paper, we consider CVD as clinical stroke or myocardial infarction, and the identification and treatment of individuals at high CVD risk among those who have not yet developed overt CVD) as a means to prevent or delay the occurrence of overt CVD.

Persons who have already developed CVD need to be treated to both improve their immediate prognosis and reduce the occurrence of further acute CVD events. Clinical treatment of acute CVD can be very effective, e.g. coronary revascularization (coronary artery bypass surgery) or percutaneous coronary intervention), but these interventions tend to be complex and costly. Because persons who have already developed clinical CVD have a very high risk of developing further events, it is a priority to identify them in order to provide long-term clinical management to reduce their CVD risk.

The optimal balance between population and high-risk strategies differs according to epidemiological and resource situations in different populations. However, any program devoted to CVD prevention and control has to include a mix of both high-risk interventions (aimed at providing cost effective treatment to selected persons at high risk of CVD, or with CVD) and population wide interventions (aimed at reducing the levels of upstream CVD determinants in the entire population). Population and high-risk strategies mutually reinforce each other, e.g. those treated for CVD conditions may be inclined to support public health interventions, while implementation of public health interventions help sensitize the individuals about the need to adopt healthy behaviours and take long-term treatment when indicated.

In high-income Western countries, the age-standardized CVD mortality rate has decreased by more than 70 % in the past four decades. Approximately 50–60 % of this reduction has been attributed to population-based interventions (i.e. reduction of CVD risk factors in the population through public health measures targeting the entire population) and 40–50 % to clinical management at the individual level [9, 10]. The substantial contribution of the high-risk strategy to reduce CVD morbidity and mortality has also been observed in LMICs, for example in Brazil [11, 12].

### Priority interventions

There is now consensus across the globe on the need to address priority NCDs in LMICs. The World Health Assembly (WHA) has agreed on 9 voluntary global targets for the prevention and control of NCDs, including a 25 % relative reduction by 2025 in premature mortality (age 30–70) from CVD, cancer, diabetes, or chronic respiratory diseases from the 2010 baseline (Table 1).

**Table 1:** Main targets for intervention to prevent main NCDs, including CVD, to be achieved by 2025 as compared to baseline in 2010

Mortality and morbidity	1	25% reduction in mortality from CVD, cancer, diabetes, or chronic respiratory diseases
Behavioural risk factors	2	10% reduction in harmful use of alcohol
	3	10% reduction in the prevalence of insufficient physical activity
	4	30% reduction in salt intake
	5	30% reduction in the prevalence of smoking in adults
Biological risk factors	6	25% relative reduction in the prevalence of raised BP
	7	0% increase in the prevalence of diabetes and obesity
National systems response	8	At least 50% of eligible people receive drug therapy and counselling (including glycemic control) to prevent heart attacks and strokes
	9	At least 80% availability of affordable basic technologies and essential medicines for NCDs in both public and private facilities

*NCD* noncommunicable diseases, *CVD* cardiovascular disease

Adapted from WHO [5]

Cost effective, affordable and scalable interventions, both at population-wide level and in high-risk groups, are described in the World Health Organization (WHO) Global NCD Action Plan, 2013–2020 (Table 2) [5, 13] and in the 2014 NCD Status Report [14]. These interventions were determined through both technical (expert reviews) and political (WHO Member States) consultations. Prioritizing cost-effective interventions is important to maximize public health gain within often very limited resources [15].

**Table 2:** Cost effective interventions for the prevention and control of NCDs as reported in the WHO Global Action Plan 2013–2020

*Clinical*	Risk factor/disease (DALYs in millions; % global burden)	Interventions (bold are ‘best buys’, others are ‘good buys’)	Averted burden	CE	Implementation cost	Feasibility *(health system constraints)*
	Tobacco use (>50m DALYs;3.7% global burden)	Raise tax on tobacco	Combined effect:	CE	Very low cost	Highly feasible; strong framework (FCTC)
		Ban tobacco advertising	25–30 million DALYs (>50% tobacco burden)			
		Ban smoking in public/work places				
		Health warning on danger of smoking				
C		Offer counselling to smokers		Quite CE	Quite low cost	Feasible (PHC)
	Harmful use of alcohol (>50m DALYs; 4.5% GB)	Restrict access to retailed alcohol	Combined effect:	Very CE	Very low cost	Highly feasible
		Enforce bans on alcohol advertising	5–10 m DALYs (10–20% alcohol burden)			
		Raise taxes on alcohol				
		Enforce drink-driving laws		Quite CE	Quite low cost	Intersectoral Feasible (PHC)
		Offer brief advice for hazardous drinking				
	Unhealthy diet (15–30m DALYs; 1–2% GB)	Reduce salt intake	Salt reduction:	Very CE	Very low cost	Highly feasible
		Replace transfat with polyunsaturated fat	5 m DALYs			
		Promote public awareness about diet				
		Restrict marketing of food and beverages to children	NA	Very CE	Very low cost	Highly feasible
		Replace saturated fat with unsaturated fat				
		Manage food taxes and subsidies				
		Provide health education in worksites		Less CE	Quite low cost	Highly feasible
		Promote healthy eating in schools				
C		Offer counselling in primary care		Quite CE	Higher cost	Feasible (PHC)
	Physical inactivity	Promote physical activity (mass media)	NA	Very CE	Very low cost	Highly feasible
	(>30m DALYs;2.1% GB)	Promote physical activity (communities) Support active transport strategies		Not assessed	Not assessed globally	Intersectoral action
		Promote physical activity in worksites		Quite CE	Higher cost	Feasible (PHC)
		Promote physical activity in schools		Less CE	Higher cost	Feasible
C		Offer counselling in primary care				
C	CVD and diabetes (170 m D; 11% GB)	Counselling & multidrug therapy for CVD and diabetes if 10-year risk of CVD ≥30%	60 m DALYs (35% CVD burden)	Very CE	Quite low cost	Feasible (PHC)
C		Aspirin for acute myocardial infarction	4 m (2% CVD B)			Feasible (PHC)
C		Multidrug therapy if 10-year risk of CVD ≥20%	70 m (40% CVD B)	Quite CE	Higher cost	Feasible (PHC)

*Abbreviations*: *C* clinical intervention (i.e. all others are public health interventions), *B* burden, *CA* cancer, *CE* cost effective, *CVD* cardiovascular diseases, *DALY or D* disability adjusted years of life lost, *FCTC* framework convention on tobacco control, *GB* global burden, *m* million, *NA* not available, *PHC* primary health care

Interventions in bold/blue are very cost effective (“best buys”), i.e. generate an extra year of healthy life for a cost that falls below the average annual income or gross domestic product per person

High-risk interventions are relevant for four of the 9 voluntary global targets: 1) 25 % relative reduction in the prevalence of blood pressure levels; 2) halting the rise of diabetes and obesity; 3) at least 50 % of eligible people receiving drug therapy to prevent heart attacks and strokes; and 4) 80 % availability of the affordable basic technologies and essential medicines to treat major NCDs. A set of 25 indicators to monitor progress toward the 9 targets has also been agreed by the WHA.

**Fig. 2:**
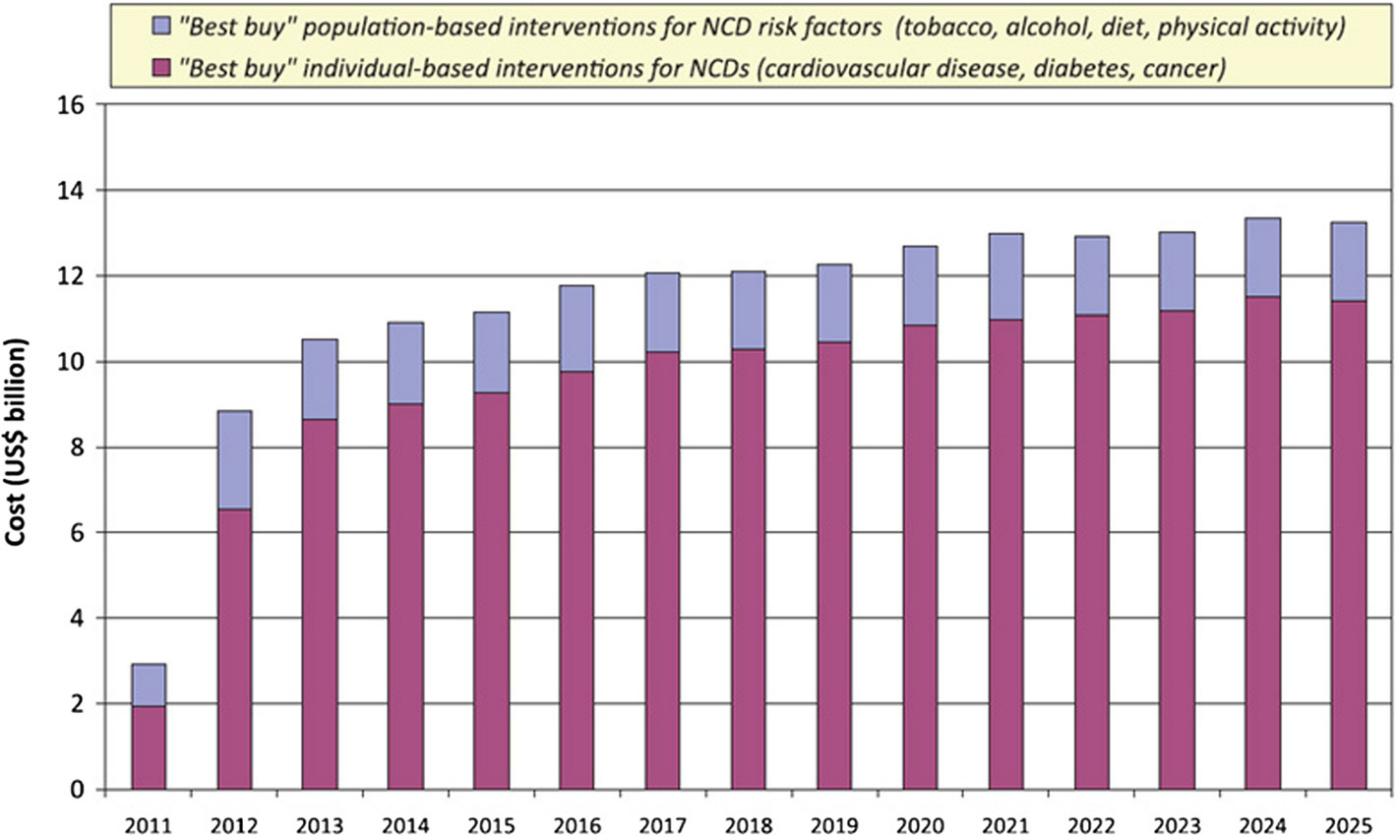
Estimated cost of scaling up best buy interventions to prevent NCDs in LMICs. Reproduced with permission from WHO [16]

**Fig. 3:**
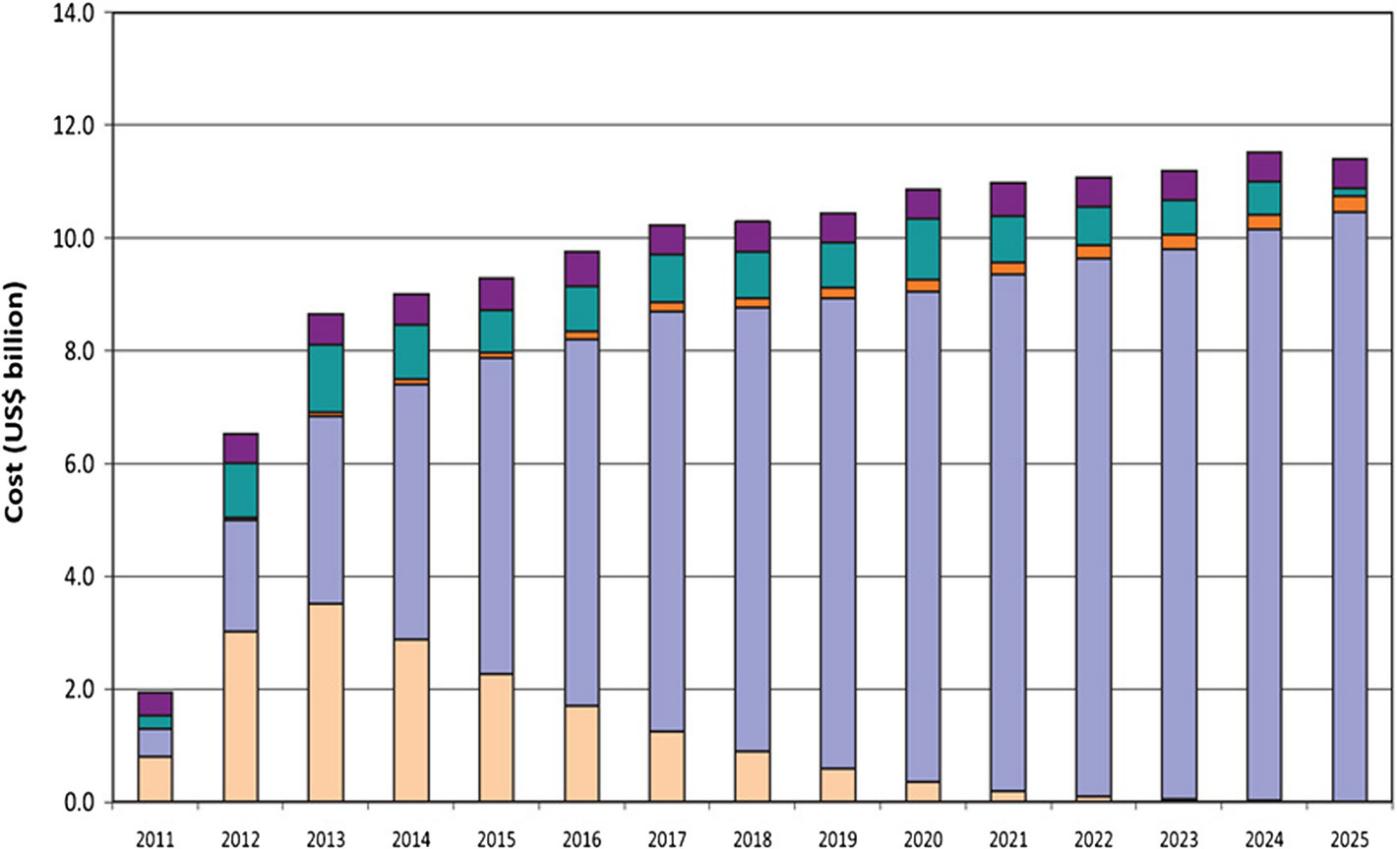
Estimated cost of scaling up high risk interventions to prevent CVD and other NCDs in LMICs. Reproduced with permission from WHO [16]

It has been estimated that implementing a package of very-cost effective, or “best buy”, population and high-risk strategic interventions for the prevention and control of the four principal NCDs would cost US$ 0.88, US$ 1.45 and US$ 2.91 per capita per year for low-income countries, lower middle-income countries and upper middle-income countries, respectively, which respectively spend US$ 22, US$ 74, and US$ 412 for total health expenditure per capita annually [16]. High-risk strategies would account for the largest share of the cost of the full package (Fig. 2) and treatment for persons at high CVD risk would account for the largest share of resources for high risk strategies for the main NCDs (Fig. 3) [16]. This underlies the need to carefully design high-risk strategies targeting persons at high CVD risk, particularly in LMICs.

### Assessing CVD risk

The majority of CVD guidelines are from high-income countries and focus on single risk factors with regards to prevention of CVD, rather than using an absolute risk approach. For example a number of guidelines suggest initiating anti-hypertensive medication for all persons with blood pressure ≥140/90 mmHg, even where the risk of CVD is low [17, 18]. However, when interventions involve potential long term treatment at the individual level, and implicitly significant resources, it is important to develop strategies that can maximize the numbers of CVD events averted and minimize the numbers of persons who need to be treated. This is important for all countries, but particularly for LMICs with their limited resources. CVD risk scores have been developed and are based on a limited number of main CVD risk factors (e.g. age, sex, hypertension, smoking, cholesterol, diabetes). A main principle underlying all CVD risk scores is that lowering blood pressure, or lowering blood cholesterol, provides similar relative risk reduction at all levels of baseline CVD risk, but progressively greater absolute risk reduction as the baseline CVD risk increases [19–23]. As a result, it is most efficient to provide treatment to those persons with the highest total CVD risk [24]. Providing treatment to individuals with a risk for developing fatal or non-fatal CVD larger than 20-30% in the next 10 years is typically considered a very cost-effective or a “best buy” intervention to reduce CVD in LMICs. A “best buy” intervention generates an extra year of healthy life for a cost that falls below the average annual gross domestic product [GDP] income per person. Risk scores are also calibrated to account for the background CVD risk in a particular population, which underlies that different risk scores have been developed in different regions.

Nevertheless, because the levels of risk factors tend to track over time in a same individual (e.g. youths with high blood pressure are likely to become adults with high blood pressure or inversely, adults with high blood pressure are likely to have had high blood pressure during their youth), some experts maintain that intervening at an earlier stage when the overall risk is only intermediate (which often means at an earlier age and/or at a lower total CVD risk) would help prevent the transition from a “moderate” to a “high” risk of CVD and reduce the number of treatment failures that can occur when treatment is initiated at the high risk stage [25–27]. Furthermore, there is some evidence to treat high risk individuals to more stringent targets (e.g. BP levels <120/80 mmHg) [28], and to extend treatment in primary prevention to individuals at lower CVD risk [29] to achieve larger CVD prevention and sustain CVD reduction over time, although the recent trends to extend treatment to increasingly low risk persons raise a number of issues [30].

A further concern is that despite the fact that CVD risk scores have good accuracy in predicting CVD risk [31], prediction of future CVD events is less reliable at the individual level [32]. Large numbers of CVD events in the population arise from the large numbers of persons with only low or moderate CVD risk. For example, more than 50 % of all CVD events in the UK arise from persons with CVD risk lower than 10 % using the QRISK2 score [33]. Furthermore, CVD risk scores have not been developed from actual data in most LMICs because of a lack of population-based cohort studies, and several issues must be considered when calibrating risk scores from one population to others [34], e.g. from high income countries to LMICs.

### CVD risk charts in LMICs

WHO has issued CVD prediction charts to assess the 10-year risk of fatal or non-fatal CVD that can be applied in different regions of the world [35–38]. The CVD score requires information on a person’s age, sex, smoking status, diabetes status, blood pressure and blood cholesterol. Risk charts also exist when levels of cholesterol cannot be measured [39]. In the WHO PEN package, a mixed approach to high CVD risk is proposed [40]. CVD medications are indicated for persons with very high CVD risk (i.e. 10-year CVD risk ≥30 %), or for persons with blood pressure ≥160/100 mmHg alone, or with cholesterol ≥8.0 mmol/l alone (who may not necessarily have a high total CVD risk).

Table 3 shows the estimated numbers of persons who would need to be treated to prevent CVD and the related costs, according to different high-risk scenarios. These estimates are based on the distribution of CVD risk factors assessed in a population-based survey of CVD risk factors in the Republic of Seychelles and are limited to the sole costs of medications related to treatment of high CVD risk [41]. Cost estimates are based on generic drugs procured mainly from India (i.e. around 10 times less expensive than in western countries) and do not account for acute care of incident or prevalent CVD, medical visits and biological or other examinations.

**Table 3:** Estimated impact and cost of different high risk strategies to prevent CVD in the Seychelles based on data in 2004

Treatment strategy	No. eligible to treat	Number of CVD events averted	Number needed to treat to avoid 1 CVD event	Total cost of medications (in US$ millions)	Cost of medications (US$) to avert 1 CVD event
BP ≥140/90 mmHg	44′899	127	354	1.84	14′534
Total cholesterol ≥6.2 mmol/l	28′317	39	727	1.24	31′831
High BP or high cholesterol	59′741	157	379	3.89	24′678
Risk ≥10%	10′837	137	79	1.03	7′499
Risk ≥20%	5′114	92	56	0.49	5′291
Risk ≥20%, BP ≥160/100, TC ≥8.0	20′653	147	140	1.96	13′307
Current situation	37′667	103	366	2.45	23′789

Adapted from Bovet et al [41]

### Who should be assessed for CVD risk?

Screening for CVD risk implies assessing individual CVD risk factors included in the CVD risk score (Table 4) [42–46]. However, because assessing CVD risk can involve lifelong treatment, screening strategies in all countries, and particularly in LMICs, must take into account available resources and competing needs. Screening of CVD risk may best achieved using opportunistic screening of selected CVD risk factors at the primary health care level, considering that a majority of the population will seek health care at some point of time. Guidelines should be adapted to local circumstances and specify who should be screened, for what, at which age, and at which time intervals. In many countries, there are pressures from the private sector, including private health care systems, which encourage screening of a variety of non essential CVD markers [47]. WHO has published a set of tools for the assessment and management of CVD risk for the prevention of heart attack and stroke in primary care, including hypertension and diabetes. The protocol considers several conditions, including age; tobacco use; increased waist circumference; known hypertension; known diabetes; history of premature CVD in first degree relatives; and history of diabetes or kidney disease in first degree relatives [40]. Of note, identifying individuals with diabetes is useful both to assess CVD risk (diabetes is a risk factor that doubles an individual’s total CVD risk) and for targeted early detection and treatment of diabetes *per se*, as diabetes also is a disease incurring complications not related to CVD. WHO is currently developing guidelines on screening for CVD risk and diabetes.

**Table 4:** Recommendations of the U.S. Preventive Service Task Force (USPSTF) for the screening of hypertension, dyslipidemia and diabetes in adults

Condition	Recommendations
High blood pressure	Recommendation to screen for high blood pressure in adults 18 and over.
Abnormal blood lipids	Recommendation to screen men aged 35 and older for lipid disorders;
	Recommendation to screen women aged 45 and older for lipid disorders if they are at increased risk for coronary heart disease.
Diabetes	Recommendation to screen for abnormal blood glucose and type 2 diabetes mellitus in adults who are at increased risk for diabetes.

Adapted from different recommendations from USPSTF [43–46]

### Management of persons with CVD risk at primary health care level in LMICs

Guidelines on screening for CVD risk need to be developed alongside guidelines on how those persons identified at risk should be managed. Guidelines should include evidence-based interventions which are affordable and feasible for a particular environment. The PEN package tools provide specific guidance for the management among patients with the main NCDs that can be used in low resources settings for both persons with CVD (heart attack and stroke) and those at high risk of CVD [40]. Management for both primary and secondary prevention of CVD needs to have a strong focus on risk factor reduction through both a healthier lifestyle and medications to control blood pressure, blood lipids, and diabetes. Further description of the management of CVD and its risk factors is beyond the scope of this review, particularly taking into account that clinical management of acute CVD events and those at high risk CVD risk will largely depend on resources available; further information is available elsewhere [42, 48]. Of note, a number of effective interventions to reduce CVD risk do not require drug therapy [42], e.g. advising smokers to quit [49] or encouraging people to adopt a healthy diet (e.g. calorie intake and salt reduction) and regular physical activity [50].

### Challenges related to assessment and management of high CVD risk

#### Overdiagnosis

A major caveat when assessing a CVD risk in the population is avoiding overdiagnosis [51]. Overdiagnosis can result in inappropriate treatment that can be both hazardous to the patients and a waste of scarce resources. In one study in Tanzania, only half of all those who had high blood pressure on a first reading (≥160/95 mmHg, i.e. at a level requiring treatment irrespective of total CVD risk) still had high blood pressure (≥140/90 mmHg) at a fourth medical visit several weeks later, with no treatment given in the interval [52]. These findings are explained by two distinct phenomena. The first mechanism is the well-known “regression to the mean”, i.e. the fact that a number of measurements that are at extreme values (high or low) on a first measurement will tend to move toward values closer to the mean value over subsequent measurements. This problem underlies the need to measure risk factors (particularly BP and blood glucose) on several different days before a definite diagnosis is done.

The second mechanism is the “white coat effect” whereby blood pressure measured by a doctor is artificially high because of anxiety related to the procedure. Indeed, blood pressure readings tend to be lower if they are self-measured or measured by other health care staff. If patients are started on treatment with an erroneous diagnosis of hypertension, a subsequent decrease in blood pressure can be erroneously attributed to antihypertensive care and treatment may be continued indefinitely. In all countries, including resource limited settings, home blood pressure monitoring (e.g. self measurement at home of blood pressure for a few days using an electronic monitoring device) can be a useful strategy to reduce false positive hypertension cases when considering the high cost of overdiagnosis, which may result in unnecessary treatment for many years and potential harmful side effects.

#### Adherence to treatment

Low adherence to treatment is another important challenge. Adherence as low as 50 % was found for treatment of hypertension and other CVD risk factors in both high and low income countries [53–55]. In Dar es Salaam (Tanzania), only 30 % of those diagnosed with hypertension were accessing health care at 12 months and less that 3 % were on treatment [56]. Explanations for this pattern include out-of-pocket expenditure for health care, which makes long term treatment too expensive; the fact that health care is not a priority for people with asymptomatic conditions; and a variety of emotional and other barriers related to perception by patients of NCDs and chronic treatment [57]. Ensuring that a patient has sufficient understanding of his/her CVD condition, the underlying causes, and the reasons for treatment, is important in encouraging adherence to long-term treatment. However health care professionals rarely have sufficient time, understand the importance of, or are rarely sufficiently incentivized to explain to their patients the need to comply with treatment. A number of other factors aimed at strengthening the entire health system have been identified to improve adherence to treatment for chronic conditions [58, 59].

#### Fixed-dose multidrug therapy

Some authors have recommended a radically simplified treatment strategy by using two simple markers (age and sex) as the basis for determining treatment and one single multipurpose fixed dose drug combination to lower blood pressure, blood cholesterol and aspirin ( i.e., the “polypill”) [60]. According to this strategy, a fixed-dose combination medication taken by all males aged ≥55 years, irrespective of their levels of other CVD risk factors, could reduce CVD by more than 80 %. Proponents of this approach highlight that this strategy has the potential to minimize the need for diagnostic testing, reduce requirements for medical follow-up, simplify treatment guidelines, enable greater task sharing for health care delivery, and enable procurement of drugs at lower costs [61]. There is no definite evidence to support broad use of fixed-dose combination therapy as yet, and efficacy, long-term risks, sustainability, and cost effectiveness of this strategy remain to be established before considering widespread use of fixed-dose combinations but evaluations are ongoing [62]. In any case, any use of a polypill should not undermine comprehensive public health approaches to NCD prevention and control and efforts to strengthen health systems in LMICs.

### Strengthening health systems for management of NCDs

The identification of CVD risk factors, and subsequent management when needed, require a strong and sustainable health system covering the whole population. Health systems in many LMICs need rapid orientation towards care of chronic conditions, including CVD and other NCDs, an area that has hitherto been neglected. This requires strengthening the health system across all its dimensions. A primary task is the appropriate education of the health workforce, both in clinical care and in public health, in order to improve the understanding of chronic diseases, including screening and long-term follow up. Other areas needing strengthening include service delivery, health information systems, access to essential medicines, sustainable financing, and leadership and accountable governance [63].

The focus for NCD prevention and control in all countries needs to be in primary care, as this is where interventions are most cost-effective and feasible, and can reach the largest number of people. Countries therefore need to ensure that their health sector strategies articulate costed plans for scaling up the health system response to NCDs, with particular emphasis on primary health care. An effective primary care response requires the training of health professionals in the diagnosis and management of CVD and its risk factors; the production and availability of concise and locally relevant guidelines on priority cost-effective interventions for CVD risk reduction [64]; the development of registries and information systems allowing easy follow up of patients’ CVD risk parameters over time (as well as records of informed preferences of “engaged patients” about their treatment); availability of simple and reliable equipment for assessing CVD risk (mainly hypertension, diabetes and dyslipidaemia); sustained availability of affordable essential priority medications for CVD risk reduction (which may include as few as a dozen of drugs for reducing CVD risk); and task shifting/task sharing so that management and/or follow-up of patients at high CVD risk (e.g. patient with well controlled hypertension and/or diabetes) can be performed by health professionals other than doctors [65–67]. A recent trial showed that a simplified cardiovascular management program conducted by community health workers at primary health care level improved quality of care and clinical outcomes in resource-poor settings in China and India compared to usual care [68].

More generally, management of NCDs in LMICs is a multi-billion dollar market for pharmaceutical and allied industries. It is therefore important that good governance, continued monitoring, involvement of the civil society, and adequate regulatory frames are set up so that treatment and prevention of CVD and other NCDs are not solely left to commercial interests [69–72].

### Access to health care

Treatment costs are often paid out-of-pocket in LMICs and the cost of monthly treatment are often a significant proportion of household income [73]. Systems requiring direct payment at the point of care prevent millions of people in the world from accessing services and can result in financial hardship, and catastrophic health expenditure which can drive individuals and families into poverty [74]. It is crucial therefore to ensure that wherever possible generic medications are used for the treatment of CVD. This emphasizes the critical importance of efficient procurement channels for medicines and diagnostic supplies in LMICs [69, 75], and ensuring that essential drugs are free of charge [76]. Universal health coverage would be a major step forward in ensuring that those with CVD and/or at risk of CVD have access to effective, affordable and accessible health care [77, 78]. However, unframed improved access to health care can create a number of distinct pressures that further prioritise curative clinical services at the expense of population-level health promotion, prevention, and action on social determinants of health, with a potential for less equitably distributed benefits [68].

## Conclusions

The high-risk strategy to reduce CVD risk in LMICs is an important component of any program for CVD risk reduction in LMICs. High risk strategies need to be carefully designed to maximize the numbers of fatal and non-fatal heart attacks and strokes averted while minimizing the numbers of persons needing treatment. Concentrating health care on those with high total CVD risk, as assessed by using CVD risk scores, enables this to be done. Because of the enormous number of people at high CVD risk in LMICs it is essential that care for high CVD risk among asymptomatic individuals is centred on primary care, with secondary care being available for the acute management of heart attack and stroke.

The increasing burden of NCDs in LMICs and the long-term management of CVD and its risk factors mean that health systems in LMICs need to be rapidly orientated around patient-centred integrated care for the management of NCDs [12, 79]. Valuable lessons can be learnt from HIV/AIDS care in LMICs [80]. Among the many measures to strengthen health systems, a number of specific issues are particularly relevant to the management of persons at high CVD risk, including simplified treatment schemes, task sharing, effective procurement of affordable medications, and universal access to health care.

In addition to an effective health system response, the reduction of CVD and other NCDs requires multisectoral population-based interventions to reduce their underlying determinants.

## Abbreviations

CVD: cardiovascular disease
LMICs: low and middle income countries
NCD: non communicable diseases
